# Evaluating health-related quality of life in Ethiopia: systematic review and meta-analysis of EQ-5D-based studies

**DOI:** 10.3389/fepid.2024.1455822

**Published:** 2024-11-01

**Authors:** Tenaw Baye Tarekegn, Desye Gebrie, Abebe Tarekegn Kassaw, Abebe Dagne Taye, Fentaw Girmaw, Getachew Ashagrie

**Affiliations:** ^1^ Department of Pharmacy, College of Health Science, Woldia University, Woldia, Ethiopia; ^2^ Department of Pharmacy, College of Health Science, Debre Markos University, Debre Markos, Ethiopia

**Keywords:** EQ-5D, Ethiopia, quality of life, meta-analysis, systematic review

## Abstract

**Background:**

Health-related quality of life (HRQoL) is crucial for understanding how health conditions impact overall well-being. The EuroQol-5 Dimension (EQ-5D) is a widely used tool for measuring HRQoL across diseases. In Ethiopia, this tool has been employed to assess HRQoL across various healthcare settings. This study aims to summarize EQ-5D-derived health outcomes in Ethiopian populations and identify key determinants influencing these outcomes.

**Methods:**

A systematic search of PubMed, Embase, and Scopus was conducted through May 2024, with no publication date restrictions, focusing on HRQoL and EQ-5D instruments in Ethiopian populations. Grey literature searches were also performed using Google's Advanced Search. Cross-sectional studies across various diseases were included. Data were extracted by two independent reviewers, and pooled mean EQ-5D utility and EQ-5D visual analog scale (EQ-VAS) scores were calculated using a random-effects model in STATA software version 17. Study quality was evaluated using the Agency for Healthcare Research and Quality (AHRQ) checklist, and heterogeneity was assessed using the *I*² statistic.

**Results:**

Fourteen cross-sectional studies involving 5,639 patients from 2019 to 2024 in Ethiopia were analyzed. Health utility values varied across diseases, with pain/discomfort and anxiety/depression being the most commonly affected dimensions. The pooled EQ-5D utility for HIV patients was 0.88, and the EQ-VAS score was 76.59. For diabetes mellitus (DM) patients, the pooled utility was 0.78, and the EQ-VAS score was 69.36. For COVID-19 patients, the pooled utility was 0.86, and the EQ-VAS score was 74.56. Cancer patients had a pooled EQ-VAS score of 67.87.

**Conclusion:**

The EQ-5D is a reliable tool for measuring HRQoL in Ethiopian patients across various diseases. The study's pooled EQ-5D scores provide valuable insights for future economic evaluations in the Ethiopian healthcare system.

**Systematic Review Registration:**

https://doi.org/10.1136/bmjopen-2024-085354, PROSPERO (CRD42024505028).

## Introduction

Health-related quality of life (HRQoL) is a multidimensional concept that reflects an individual's perception of their overall health, taking into account various aspects of physical, mental, and social health (1). It is a critical measure in public health and healthcare research as it provides valuable insights into the impact of diseases, medical interventions, and socio-economic factors on individuals’ lives (2, 3). Several tools are used to measure HRQoL and calculate utility values for cost-effectiveness analyses in health economics. These include generic instruments like the EuroQol-5 Dimension (EQ-5D), the Short Form-6 Dimension (SF-6D), and the Health Utilities Index (HUI), among others. Each tool has distinct features and strengths, making them suitable for different types of studies and populations (4, 5). For instance, the SF-6D is derived from the widely used SF-36 and SF-12 surveys, offering a broader assessment of health dimensions, while the HUI provides detailed measures on attributes such as vision, hearing, and dexterity (6).

The EQ-5D, developed by the EuroQol Group, has emerged as a widely used tool for quantifying HRQoL, offering a standardized and comprehensive assessment across five key dimensions: mobility, self-care, usual activities, pain/discomfort, and anxiety/depression (7). The original version of the EQ-5D, known as the EQ-5D-3l, was introduced with three response levels for each of the five dimensions: no problems, some problems, and extreme problems. To address concerns regarding its limited sensitivity, the EuroQol Group later developed the EQ-5D-5l, which introduced five response levels: no problems, slight problems, moderate problems, severe problems, and extreme problems. This expanded scale enhances the instrument's ability to detect subtle changes in health status and reduces ceiling effects, making it a more sensitive tool for HRQoL measurement (8). Additionally, both versions of the EQ-5D include a visual analog scale (EQ-VAS) that asks respondents to rate their overall health on a scale from 0 (worst health) to 100 (best health) (7).

The EQ-5D has been translated into various languages and validated for use in over 170 countries (9). Its flexibility, simplicity, and cross-cultural applicability have made the EQ-5D a preferred tool for measuring patient-reported outcomes across various healthcare settings, including primary care, hospitals, and population health surveys, and it is widely used to assess HRQoL in patients with a variety of conditions, such as diabetes, hypertension, cardiovascular diseases, cancer, and HIV/AIDS. Its application covers many countries, such as China, where it has been used to evaluate HRQoL in patients with diabetes mellitus (10) and hypertension (11), as well as estimate the minimally important difference in elderly populations. It is also employed in systematic reviews covering various diseases (12). In the USA, population norms have been established through both face-to-face and online samples (13), while other countries like Singapore (14) and Japan (15) have developed their own population norms using EQ-5D-5l, specified to local preference weights. Additionally, the EQ-5D serves as a standard tool in the economic evaluation of healthcare interventions in the UK and the USA (16), China (17), India (18), Egypt (19), Brazil (20), and Australia (21). It generates utility values for calculating quality-adjusted life years (QALYs), a key metric in health economics (22).

The EQ-5D has also been extensively used in developing countries, including Ethiopia, where its simplicity is particularly valuable for large-scale population studies, even in resource-limited settings. Using the standardized process advised by the EuroQol Group, the EQ-5D-5l has been validated for use in Ethiopia and translated into Amharic, the country's official language (23). However, the use of the EQ-5D-3l in Ethiopia has been limited, mainly due to the absence of a designated reference value set. In the absence of country-specific reference values, studies in Ethiopia have relied on general population tariffs from other countries, particularly Zimbabwe (24). Ethiopia faces health challenges including infectious diseases like HIV and TB, non-communicable diseases, and maternal health issues, all affected by socio-economic factors, geographical disparities, and cultural diversity (25). Understanding HRQoL is vital for designing targeted interventions, resource allocation, and policy development. The EQ-5D, with its sensitivity to detecting small changes in health status, provides an important opportunity to assess HRQoL in Ethiopian patients with a variety of health conditions. Moreover, economic evaluations based on utility values derived from the EQ-5D have been used to inform health policy and decision-making in the country (26).

Despite the increasing recognition of HRQoL as a critical outcome measure globally, no comprehensive review has synthesized the evidence from Ethiopian populations using the EQ-5D instrument. This study aimed to fill that gap by conducting a systematic review and meta-analysis to quantitatively synthesize existing literature, providing pooled utility scores across different health conditions. The study also explored factors influencing HRQoL, including socio-demographic characteristics, health status, and specific diseases. Additionally, it critically assessed the methodological quality of the available studies, offering recommendations for future research in HRQoL measurement in Ethiopia.

## Methods

### Searches and data sources

A systematic review was done to assess HRQoL in Ethiopia, using studies that employed the EQ-5D tool. The review followed the Preferred Reporting Items for Systematic reviews and Meta-Analyses (PRISMA) 2020 statement (27), which provide clear instructions for conducting and reporting systematic reviews in healthcare (Supplementary Table S1). The protocol had been registered on PROSPERO, ID: CRD42024505028**.**

A literature search was conducted using PubMed, Embase, and Scopus. The searches used the following keywords and Medical Subject Headings (MeSH) alone and in combination, including: “quality of life,” “QoL,” “HRQoL,” “EQ-5D,” “EQ-5D-3l,” “EQ-5D-5l,” “EuroQol,” and/or “Ethiopia” as described in Supplementary Table S2. Grey literature searches conducted through Google's Advanced Search supplemented database searches. The reference lists of included research were checked, and relevant articles were identified to ensure a sufficient body of literature. The search included articles published up to May 2024, without any restrictions on publication dates, and the final search was conducted on May 15, 2024.

### Eligibility criteria

#### Study inclusion criteria

Studies were required to meet the following criteria in order to be included in the review:

• Population: people with all type of diseases with or without comorbidities;
• Intervention: none;
• Comparator: none;
• Outcomes: EQ-5D utility scores, EQ-VAS scores;
• Study design: cross-sectional studies;
• Setting, country: Ethiopia, all health settings

#### Exclusion criteria

The following studies were excluded from the review:

1. Studies without a full-text version available in English;
2. Letters to the editor, notes, comments, news or newsletter items, doctoral theses, conference proceedings (not published in a peer-reviewed journal) and meeting minutes;
3. Studies focused on the general population;
4. Longitudinal studies or effects evaluation studies of different interventions;
5. Studies that reported only synthetic utilities of multiple diseases, non-EQ-5D utilities, or no utilities; and
6. Studies not related to HRQoL.

### Study selection

**Figure 1:**
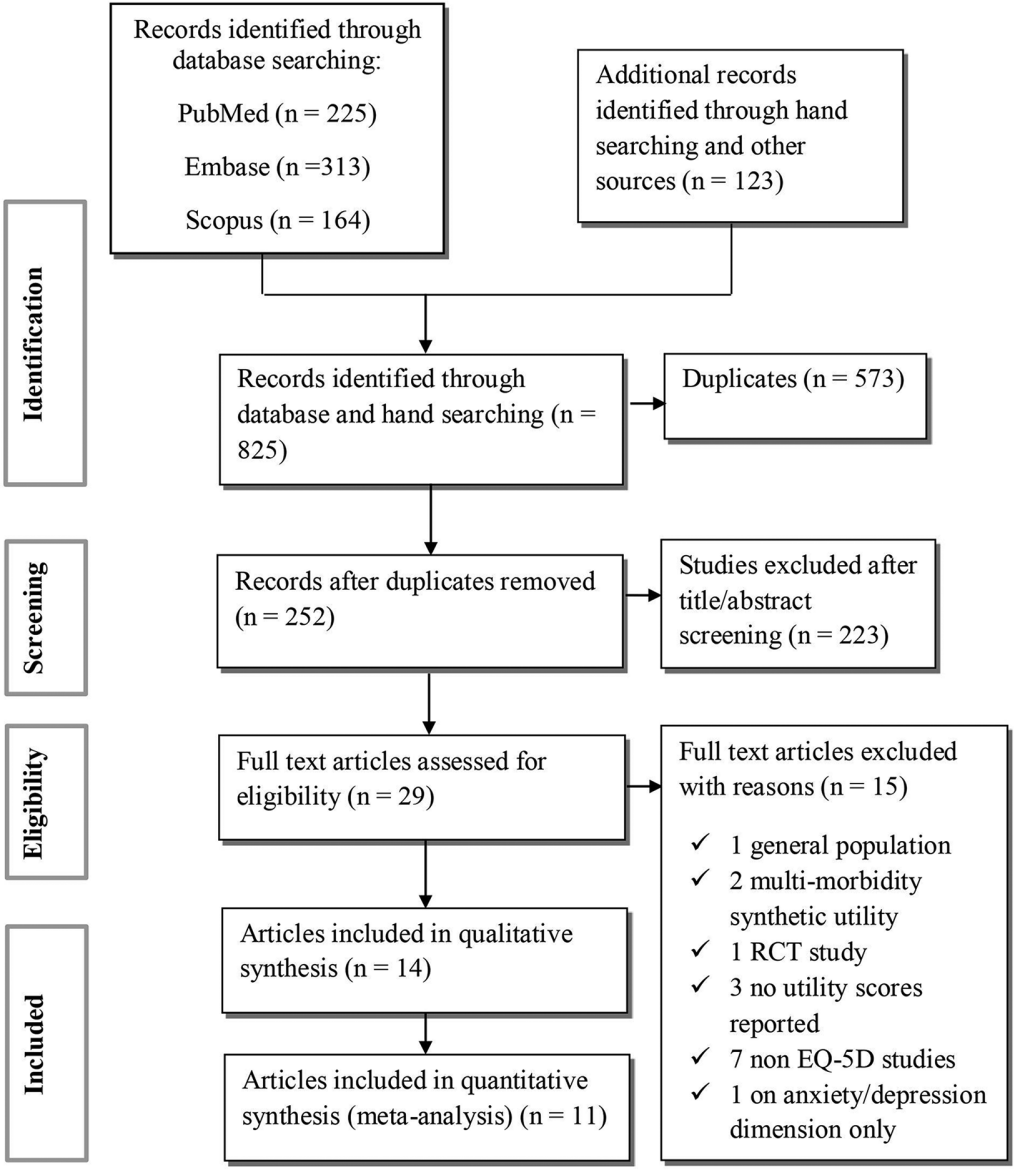
Preferred reporting items for systematic review and meta-analysis protocols (PRISMA) flowchart.

All studies identified from different search databases were transferred to EndNote 7, and duplicates were removed. Firstly, three authors (TB, FG, and GA) independently assessed the title and abstract of all studies for eligibility. Secondly, the same authors retrieved the full texts independently. Any disagreements were discussed with the other author (DG, ADT and ATK) until consensus was reached concerning the eligibility of the papers to be included. The PRISMA flowchart shows the study selection process (Figure 1**)**.

### Data collection process

Data from the reports were collected using a structured Excel data extraction format. Two independent reviewers (TB and ATK) extracted data from each report, ensuring consistency and minimizing bias. The data were then verified by two additional reviewers (GA and FG), who cross-checked the data for accuracy. Any disagreements or discrepancies between the reviewers were discussed and resolved through consensus with the involvement of third-party reviewers (DG, ADT). All data were obtained directly from the published reports.

### Data items

The main outcomes of interest were the EQ-5D utility scores, reflecting overall health-related quality of life, and EQ-VAS scores, which represent self-reported health status using the visual analogue scale within the EQ-5D tool. All results that matched with these outcome measures were extracted, including mean utility values and distributions reported in each study. In cases where multiple time points were reported, data from the most recent time point were prioritized. Additional data collected included participant characteristics such as age, sex, disease type, and comorbidities. Study-specific information, including author names, publication year, region of the study, sample size, and healthcare setting, was also extracted. Information related to the EQ-5D, such as the proportions of participants reporting problems in the five dimensions (mobility, self-care, usual activities, pain/discomfort, anxiety/depression), value sets used (Ethiopian or other country), and the method of administration (self-administered or interviewer-administered), was included as well. Funding sources were recorded where available, to assess potential bias. For studies with missing or unclear information, assumptions were made based on what was provided. For instance, when participant characteristics such as age or sample size were vague, the most detailed available information was used.

### Assessment of study quality and risk of bias

The quality of the included studies was assessed using the Agency for Healthcare Research and Quality (AHRQ) checklist (28) (Supplementary Table S3). This tool evaluates the methodological quality and potential bias of studies across 11 items, scored as “Yes” (fully reported), “No” (not reported), or “Unclear.” A total score of up to 11 points was assigned to each study. Studies scoring ≥8 were categorized as “high quality,” 4–7 as “moderate quality,” and ≤3 as “low quality.” Three authors (TB, FG, and GA) conducted the assessments, with disagreements resolved by consultation with DG and ATK.

### Effect measures

The primary effect measures were EQ-5D utility scores and EQ-VAS scores reported across different studies, focusing on the mean values and distribution of these scores within each population group.

### Data synthesis

The process for deciding which studies were eligible for synthesis involved grouping studies based on population characteristics, outcomes (EQ-5D utility scores and EQ-VAS scores), and study settings. Only studies that met the inclusion criteria and reported these specific outcomes were included in the synthesis. Data preparation involved cleaning and verifying extracted data for consistency. In cases of missing summary statistics, attempts were made to derive the needed data from other reported metrics, though no data conversions were required since the outcomes were uniformly reported. Results from individual studies were tabulated in an Excel sheet, summarizing EQ-5D utility scores, EQ-VAS scores, participant demographics, and study settings. These results were visually displayed using summary tables and figures to illustrate key comparisons and trends. A narrative synthesis was conducted due to the variability in study populations and outcomes.

### Outcome and data analysis

The primary outcome measure for the meta-analysis was EQ-5D utility scores, with mean EQ-VAS scores considered as secondary outcome measures. The meta-analysis combined EQ-5D utility and EQ-VAS scores data from studies that reported utility values along with standard error or deviation for a specific disease. In cases where data were reported in a single study, a narrative synthesis or systematic review of studies was conducted. When study results were reported as median and range or interquartile range (IQR), they were converted to mean and standard deviation (SD) using a standard transformation method (29). Effect sizes, represented by means of EQ-5D utility and EQ-VAS scores, were pooled.

Heterogeneity across studies was assessed using the *I*^2^ index, indicating the percentage of overall variation between articles attributed to heterogeneity rather than chance. Predefined thresholds for low and high *I*^2^ indices were set at 25% and 75%, respectively. The calculation of pooled EQ-5D utility and EQ-VAS scores for specific diseases was conducted using random-effect (Der Simonian–Laird estimator method) model (30). The meta-analysis was carried out using STATA software version 17. A statistical test with a significance level of *p* < 0.05 guided the interpretation of bias.

## Results

### Study selection

A total of 825 records were initially identified through both database and hand searching. Following the removal of 573 duplicate records, 252 unique records remained. Upon excluding 228 studies after title/abstract screening, 24 full-text articles were assessed for eligibility. Among the full-text articles, 10 were excluded for the following reasons: general population (*n* = 1) (23), use of synthetic utility for multi-morbidity (*n* = 2) (31, 32), no utility scores reported (*n* = 3) (32–34), non-EQ-5D studies (*n* = 3) (35–37), and focus only on the anxiety/depression dimension (*n* = 1) (38). Finally, 14 articles were included in the qualitative synthesis (39–52). Out of these, 11 studies were included in the quantitative synthesis (meta-analysis) (Figure 1).

A total of 5,639 patients were included, and the studies were conducted between 2019 and 2024. Notably, two studies exclusively focused on female patients (44, 45), and one study focused on males (48), while the remaining studies included participants of both sexes. The data collection methods varied, with 5 studies utilizing a self-administered mode, while the rest chose for an interviewer-administered approach. The majority of studies (57.3%) were carried out in Addis Ababa (34, 39, 40, 43–45, 47, 48). Among the evaluated studies, 10 utilized EQ-5D-5l for assessing utility scores, while 4 studies chose for EQ-5D-3l. All of the studies utilized a cross-sectional design. Funding sources varied, with several studies receiving support from institutions like Addis Ababa University, the Canada Research Chair in Economics of Infectious Diseases, and the US NIH. However, a high number of studies (*n* = 6) were unfunded, and two did not report their funding sources (Table 1).

**Table 1:** Characteristics of included studies.

Author year	Region	Study period	Study design	Sample size	Female (%)	Age (SD)	Funding	AHRQ score
Sibhat et al., 2019 (44)	Addis Ababa	December 2017–February, 2018	Cross-sectional	404	100	43.94 ± 11.72	Addis Ababa University	8
Araya et al., 2020 (45)	Addis Ababa	January–June 2018	Cross-sectional	404	100	52.1 ± 10.4	Addis Ababa University	6
Kaso et al., 2022 (53)	Oromia and SNNPR	1st January 2020 and 20th October 2021	Cross-sectional	493	35.1	40.11 ± 13.1	No fund	6
Kaso et al., 2021 (51)	Oromia	July 1, 2020–March 20, 2021	Cross-sectional	398	40	41.5 (SD: 18.8)	No fund	4
Sendekie AK, et al., 2023 (41)	Amhara	April–July 2022	Cross-sectional	402	45.8	55.1 ± 10.7	No fund	7
Negash et al., 2023 (47)	Addis Ababa	May–June 2022	Cross-sectional	319	48.9	≥18	No fund	7
Gebremariam GT et al., 2022 (40)	Addis Ababa	January–June 2019	Cross-sectional	360	55.7	64.43 ± 10.61	Canada research chair in economics of infectious diseases	9
Haftu et al., 2022 (49)	Tigray	January 1, 2019–March 31, 2019	Cross-sectional	415	52.3	≥18	Not reported	5
Shimels et al., 2021 (34)	Addis Ababa	August 2020	Cross-sectional	371	37.2	≥18	Saint Paul's Hospital Millennium Medical College	8
Tegegne, 2023 (50)	Amhara	March 2018–February 2021	Cross-sectional	700	46.7	Not reported	Not reported	3
Belay et al., 2021 (39)	Addis Ababa	March, 2019	Cross-sectional	511	60.5	42 ± 11	Addis Ababa University	4
Tito et al., 2022 (43)	Addis Ababa	July–September 2021	Cross-sectional	357	65.8	49.3 ± 17.8	Canadian research chair in economics of infectious diseases	8
Belachew and Sendekie, 2023 (46)	Amhara	June–August 2022	Cross-sectional	400	56	39.79 ± 17.17	No fund	7
Iyar et al., 2024 (48)	Addis Ababa	March and June 2023	Cross-sectional	105	0	21.09 ± 7.37	No fund	7

AHRQ, Agency for healthcare research and quality; COVID-19, Corona virus 19; CVD, cardiovascular diseases; DM, diabetes mellitus; HIV, human immune virus.

### Quality of the studies

The scores on the AHRQ checklist ranged from 3 to 9 points, with an average score of 6.1 and a mode of 7 (Supplementary Table S2). Notably, only one study was rated as low-quality (50). Among the evaluated studies, 10 were classified as moderate quality, and 3 were considered high quality (Table 1). This suggests that the methodological rigor of the included studies is generally acceptable, with a considerable proportion achieving a moderate quality rating.

### Pooling of EQ-5D-5l utility and EQ-VAS scores

This study assessed health utility values and self-reported health status using the EQ-5D utility score and the EQ-VAS across various diseases. These scores were derived from studies conducted in different health settings in Ethiopia, with some utilizing other value sets like the Zimbabwe tariff. The results highlight the variability in health-related quality of life (HRQoL) across different conditions, influenced by the severity and chronicity of the diseases. Diabetes mellitus (DM), neoplasms, coronavirus-19 (Covid-19), and human immunodeficiency virus (HIV) infection were the focus of two or more studies, prompting the implementation of meta-analyses. It's important to mention that the EQ-5D utility scores of neoplasms (44, 45) were excluded from the meta-analysis due to the limited reporting of standard error/deviation, with only one study (44) providing such information. Likewise, the EQ-VAS scores for two studies on DM were excluded from the meta-analysis due to the absence of reported standard error/deviation (49) and EQ-VAS scores (47). The distributions of EQ-5D data are presented in Table 2.

**Table 2:** Summary of EQ-5D data distribution and health related quality of life (HRQoL).

Diseases		EQ-5D utility score	EQ-VAS score	Value set	Reported problems across five dimensions (%)					Mode of administration
		Mean (SD)	Mean (SD)		MO	SC	UA	PD	AD	
Neoplasm										
Sibhat et al., (44)	Breast cancer	0.80 (±0.25)	69.9 (±20.4)	Ethiopian	29	15.3	36.4	54.7	41.3	Self-administered
Araya et al., (45)	Cervical cancer	0.77 (NR)	65.7 (±20.8)	Ethiopian	63.9	22.5	69.3	83.9	60.4	Self-administered
Covid-19										
Kaso et al., (53)	Covid-19	0.90 (±0.14)	82.7 (±15.6)	Ethiopian	40.4	40.6	40.8	49.1	54.6	Self-administered
Kaso et al., (51)	Covid-19	0.69 (±0.28)	69 (±12.9)	Zimbabwe tariff	NR	NR	NR	NR	NR	Interviewer administered
Diabetes mellitus										
Sendekie AK, et al., (41)	DM 1 and DM 2	0.56 (±0.11)	56.7 (±10.1)	Ethiopian	99.2	99.3	100	100	100	Self-administered
Negash et al., (47)	DM 1 and DM 2	0.89 (±0.19)	NR	Ethiopian	25.4	17.9	27.9	45.1	30.1	Self-administered
Gebremariam GT et al., (40)	DM2	0.93 (±0.06)	80 (±7.40)	Ethiopian	60.5	37.2	34.1	67.3	43.5	Self-administered
Haftu et al., (49)	DM 2	0.73 (±0.23)	87.4 (NR)	Ethiopian	29.9	31.6	35.9	48.7	52.3	Self-administered
HIV/AIDS										
Shimels et al., (34, 42)	HIV	0.87 (±0.05)	81 (±15.0)	Zimbabwe tariff	NR	NR	NR	NR	NR	Interviewer administered
Tegegne, (50)	HIV	0.39 (±0.41)	66.2 (±17.2)	Zimbabwe tariff	NR	NR	NR	NR	NR	Interviewer administered
Belay et al., (39)	HIV	0.94 (±0.10)	80 (±14.8)	Ethiopian	16.6	8	19.3	51.3	55.2	Self-administered
CVD										
Tito et al., (43)	Cardio vascular disease	0.77 (±0.27)	68.3 (±25.9)	Ethiopian	73.4	23	61	75.4	39.6	Self-administered
Dermatological disorders										
Belachew and Sendekie, (46)	Different dermatological disorders	0.92 (±0.74)	68.9 (±24.2)	Ethiopian	28.7	36	50.2	88.5	53.2	Self-administered
Hemophilia										
Iyar et al., (48)	Hemophilia	0.78 (±0.26)	75 (±7.41)	Ethiopian	59.8	41.9	69.5	77.1	51.8	Self-administered

AD, anxiety/depression; COVID-19, corona virus 19; CVD, cardiovascular diseases; DM, diabetes mellitus; EQ-5D, euroQol-5 dimension; EQ-VAS, EuroQol-visual analogue scale; HIV, human immune virus; MO, mobility; NR, not reported; PD, pain/discomfort; SC, self-care; UA, usual-activities.

### Human immunodeficiency virus (HIV) infection

Across three different studies examining individuals with HIV, the health utility values varied between 0.39 and 0.94 (34, 39, 50). The studies reporting the highest and lowest utility values utilized the Ethiopian standard EQ-5D-5l value set (39) and the Zimbabwe EQ-5D-3l value set (50), respectively. In three studies, the EQ-5D VAS scores exhibited a range between 66.2 and 81. Belay et al. reported that the decline in utility observed in individuals with HIV was primarily attributed to challenges associated with the anxiety/depression dimension of the EQ-5D-5l (39). The quality of life for individuals living with HIV is influenced by several interrelated factors. Key determinants include female gender, older age, and low educational attainment, which are associated with poorer quality of life. Additionally, infrequent appointment attendance, non-disclosure of disease status, substance addiction, and the presence of comorbidities negatively impact well-being. Economic challenges, such as unemployment and lower household income, along with clinical factors like lower CD4 counts and polypharmacy, further exacerbate these problems. These factors highlight the complex challenges faced by individuals with HIV and the need for comprehensive strategies to enhance their quality of life.

**Figure 2:**
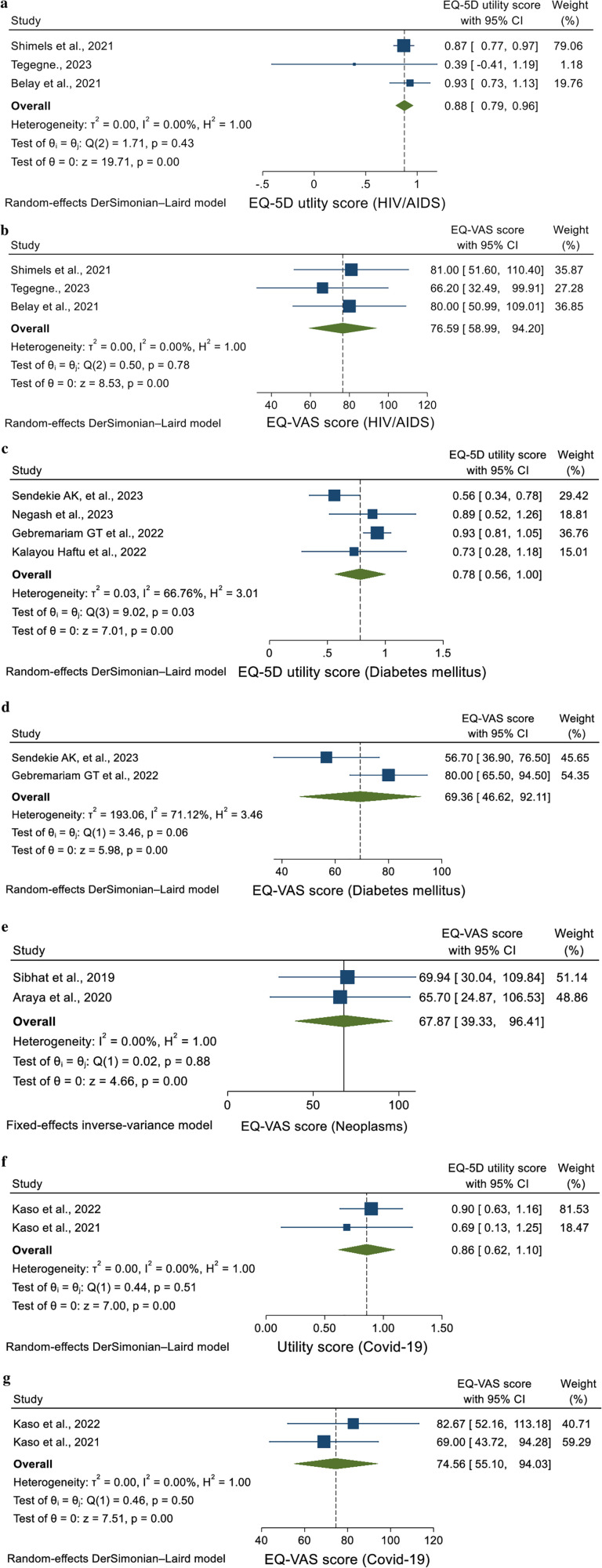
**(a)** Forest plot of the EQ-5D utility score of patients with HIV/AIDS **(b)** forest plot of the EQ-VAS score of patients with HIV/AIDS **(c)** forest plot of the EQ-5D utility score of patients with DM **(d)** forest plot of the EQ-VAS score of patients with DM **(e)** forest plot of the EQ-VAS score of patients with COVID-19 **(f)** forest plot of the EQ-5D utility score of neoplasm patients **(g)** forest plot of EQ-VAS score of patients with neoplasms.

Using a random-effect model, the pooled EQ-5D utility value for individuals living with HIV was calculated to be 0.88 (95% CI = 0.79–0.96, *I*^2^ = 0.00%, *p* = 0.43) (Figure 2a). Additionally, the pooled EQ-VAS score for these patients was 76.59 (95% CI = 58.99–94.20, *I*^2^ = 0.00%, *p* = 0.78) (Figure 2b). The findings indicated that all studies were consistent.

### Diabetes mellitus

For patients with diabetes mellitus (DM), four studies (40, 41, 47, 49) reported health utility values ranging from 0.56 to 0.93. The Ethiopian standard EQ-5D-5l value set was employed to derive both the upper (40) and lower (41) utility values. Among these studies, two specifically focused on individuals with type 2 diabetes (40, 49), while the remaining included both type 1 and type 2 diabetes patients (41, 47). It is worth mentioning that one study specifically incorporated all adult diabetes patients who were actively receiving insulin therapy (47). It is important to emphasize that while three of the studies employed the Ethiopian EQ-5D-5l value set, the study conducted by Kalayou et al. chose for the Zimbabwe EQ-5D-3l value set tool. The EQ-5D VAS scores, as outlined in three studies, showed a range from 56.7 to 87.4 (40, 41, 49). Across the five dimensions of the EQ-5D, with the exception of one study (49) where anxiety/depression was prominent, pain/discomfort emerged as the dimension with the highest frequency of reported problems. Various factors significantly influence the quality of life for individuals living with DM. These include older age, inadequate glycemic control, and an extended duration of the disease, which often leads to complications and increased body weight. Higher body mass index, a higher number of medications, and elevated blood glucose levels have been shown to correlate with a decline in quality of life. Additionally, the occurrence of comorbidities, episodes of hypoglycemia, and the requirement for insulin treatment further complicate patients’ experiences. Socioeconomic factors such as occupational status, lower income levels, prolonged waiting times for healthcare, and lower educational attainment also play a crucial role in affecting their overall well-being.

In this study using the random-effect model, the pooled utility value for patients with DM was found to be 0.78 (95% CI = 0.56–0.1.00, *I*^2^ = 66.76%, *p* = 0.03) (Figure 2c). Additionally, the pooled EQ-VAS score for these patients was 69.36 (95% CI = 46.62–92.11, *I*^2^ = 71.12%, *p* = 0.06) (Figure 2d). The heterogeneity was high, as indicated by an *I*² and *p* value.

### Neoplasms

Two studies presented health utility values for cancer patients were 0.77 and 0.80 (44, 45). The higher utility value was observed in patients diagnosed with breast cancer, both in new diagnoses and follow-ups, utilizing the Ethiopian value set (23). Conversely, the lower value was reported in patients diagnosed with cervical cancer, employing the same value set (23). In these studies, EQ-VAS scores were 65.7 and 69.94. The decline in health utility among cancer patients predominantly derived from difficulties related to the pain/discomfort dimension of the EQ-5D.

The pooled EQ-VAS score for neoplasm patients was 67.87 (95% CI = 39.33–96.41, *I*^2^ = 0.00%, *p* = 0.88). The heterogeneity was minimal, as shown by an *I*² and *p* value, using the random-effect model (Figure 2e).

### Coronavirus-19 (COVID-19)

In studies examining health utility values for individuals with Covid-19 were 0.68 and 0.90 (51, 53), with differences associated with the use of different value sets, specifically the Ethiopian EQ-5D-5l (53) and Zimbabwe EQ-5D-3l (51). The study employing the Ethiopian value set reported higher utility values, while the Zimbabwean set reported lower values. Furthermore, the EQ-VAS scores in these studies were 69 and 82.7. Both studies reported a range of comorbidities, including hypertension (HTN), DM, cardiovascular diseases (CVD), chronic obstructive pulmonary diseases (COPD), asthma, malignancy, HIV, and chronic kidney diseases (CKD). In terms of utility scores associated with these comorbidities, one study found that the highest score of 0.94 was attributed to CVD, COPD, and asthma, while the lowest score of 0.74 was reported for malignancy (53). Conversely, the second study reported the highest utility score of 0.79 for malignancy, while the lowest score of 0.59 was recorded for HTN, DM, COPD, CVD, CKD, and asthma (51). Analysis of the EQ-5D dimensions highlighted that anxiety/depression dimension significantly affected the HRQoL of Covid-19 patients. Moreover, individuals who received dexamethasone and intranasal oxygen supplementation, those with comorbidities, and those older than 55 years experienced significantly lower quality of life compared to their counterparts. Additionally, the presence of comorbidities, along with a prolonged hospital stay of more than 15 days, adversely affected their quality of life. Factors such as the patients’ health status at admission and the overall length of hospitalization further contribute to a poor quality of life.

The pooled utility value of COVID-19 patients was found to be 0.86 (95% CI = 0.62–1.10, *I*^2^ = 0.00%, *p* = 0.51), using the random-effect model (Figure 2f). Additionally, the pooled EQ-VAS score for these patients was 74.56 (95% CI = 55.10–94.03, *I*^2^ = 0.00%, *p* = 0.50) (Figure 2g). This analysis showed no heterogeneity.

### Other diseases

For CVD, dermatological disorders, and hemophilia, only one study reported the EQ-5D utility and EQ-VAS scores for patients with each disease (43, 46, 48). The study on dermatological disorders revealed the highest utility value of 0.92 (46), while patients with CVD reported the lowest value of 0.77 (43). Hemophilia patients, as per the EQ VAS score, scored the highest value of 75 (48). In all studies, the Ethiopian standard EQ-5D-5l value set was utilized to derive utility values. Across all diseases, pain/discomfort emerged as the dimension with the most frequently reported problems by patients (Table 2). Several factors are significantly negatively associated with HRQoL in patients with hemophilia, cardiovascular disease, and dermatological disorders. Key determinants include older age, prolonged duration of the disease, and the presence of comorbidities. Additionally, living in rural areas, unemployment, a history of previous hospital admissions and non-adherence to lifestyle modifications further exacerbate the decline in HRQoL. The presence of three or more cardiovascular disease risk factors also contributes significantly to poorer health outcomes.

Generally, most studies were assessed as having moderate quality, with four rated as high quality and one as low quality, indicating an overall moderate methodological quality across the studies. Utility values and EQ-VAS scores varied depending on different factors. A qualitative synthesis revealed that utility scores were generally lower in studies using the Zimbabwe tariff compared to the Ethiopian EQ-5D-5l value set. This variability in scores may be due to differences in disease severity, comorbidities, and the value sets used. For example, studies employing the Zimbabwe tariff consistently reported lower scores. Sensitivity analyses were not conducted due to a lack of comparable quantitative data. There was no clear evidence of reporting bias, though some studies did not fully report health utility values, which may have affected the overall assessment of HRQoL. The certainty of evidence was moderate for most outcomes, while confidence in health utility values was lower in studies with methodological limitations, such as small sample sizes or reliance on self-reported data.

## Discussion

The systematic review and meta-analysis conducted in this study aimed to synthesize and analyze health utility values derived from EQ-5D instruments across various diseases in Ethiopia. The findings highlighted both consistent trends and significant variations among different conditions. Across all conditions studied, pain/discomfort and anxiety/depression emerged as primary contributors to reduced HRQoL, underscoring their universal impact on patient well-being. Meta-analyses consolidated utility data from diseases reported in at least two studies. Overall, the pooled results for most diseases showed consistent findings, indicating a uniform impact on HRQoL across the broader Ethiopian population.

Health utility or QoL is a multidimensional concept that evaluates how individuals perceive their overall well-being in relation to their health and healthcare experiences (1). Assessing QoL is essential in public health and healthcare research, providing insights into how diseases, treatments, and socio-economic factors impact individuals’ lives (3). This review shows the significant impact that chronic diseases like diabetes, HIV/AIDS, TB, cancer, and others have on patients’ HRQoL in Ethiopia. These results reflect global studies, which similarly report that chronic diseases and their associated complications lead to a significant reduction in quality of life.

For example, diabetes mellitus (DM) patients in Ethiopia exhibited a pooled utility value of 0.78, with significant heterogeneity driven by factors like diabetic complications (neuropathy, nephropathy, retinopathy), comorbidities (hypertension, cardiovascular diseases), older age, and insulin therapy. This variability in HRQoL experiences is also seen in studies from other countries. In Iran, for instance, the utility score for diabetic patients was higher, around 0.83, due to better access to healthcare and lower rates of comorbidities (54). By contrast, in countries like Nigeria, where healthcare access is more uneven, utility values for diabetic patients were closer to 0.77, similar to Ethiopia (55). This emphasizes the importance of tailoring healthcare interventions based on local disease burdens and resource availability.

Patients with HIV in Ethiopia reported pooled utility values of 0.88 and EQ-VAS scores of 76.59, with no significant heterogeneity, suggesting stable HRQoL assessments. However, the study identified key factors like advanced age, lower CD4 cell counts, and comorbidities that can negatively impact QoL. In comparison, studies from Zimbabwe and Nigeria reported lower utility values for HIV patients, with averages of 0.67 and 0.72, respectively (56, 57). These differences might stem from variations in access to antiretroviral therapy (ART), healthcare infrastructure, and socio-economic conditions. Like Ethiopia, patients in these countries also experience HRQoL declines due to factors like low CD4 counts and comorbidities, but the higher heterogeneity in these studies highlights the greater variability in treatment outcomes.

In the case of neoplasm (cancer) patients, the pooled EQ-VAS score in Ethiopia was 67.87, with minimal heterogeneity. This indicates relatively uniform perceptions of health status, with pain/discomfort being a primary factor affecting HRQoL. In contrast, studies from India reported higher HRQoL utility scores, averaging around 75.0, reflecting better pain management and psychological support (58). Similarly, in Indonesia, patients reported utility values of 75.8, indicating a significant HRQoL decline due to insufficient access to effective cancer treatments and supportive care (59).

COVID-19 patients in Ethiopia reported relatively high HRQoL scores (utility value of 0.86 and EQ-VAS score of 74.56), which suggests that most patients reported better-than-expected health outcomes. This is consistent with studies from other countries like Japan, where utility values for COVID-19 patients were also relatively high (around 0.85) due to effective management of mild and moderate cases (60). However, in severely affected populations, such as those in Ghana, utility values were much lower (61), reflecting the diverse impacts of disease severity, recovery rates, and healthcare access on HRQoL outcomes. However, the impact of COVID-19 on HRQoL is largely indirect, driven by associated conditions such as respiratory complications, mental health challenges like anxiety and depression, and long-term effects like fatigue, often referred to as “long COVID.” The variability in health outcomes, differing levels of disease severity, and recovery trajectories among individuals contributed to the heterogeneity observed in HRQoL assessments (62). The review shows that patients with comorbidities, such as diabetes or hypertension, often experience a more significant reduction in their quality of life when affected by COVID-19. This finding is consistent with studies from countries like Bangladesh (63), Iran (64), and Italy (65). These studies consistently show that COVID-19 patients with pre-existing comorbidities report worse HRQoL outcomes, supporting the importance of early interventions and post-recovery support.

Limited data were available for CVD, dermatological disorders, and hemophilia, with each reporting varying utility values. Despite the differences in utility values, pain and discomfort consistently emerged as significant issues across all these conditions. This underscores the universal impact of pain and discomfort on HRQoL, irrespective of the specific disease (66). Effective pain management is essential across these diverse medical conditions to improve HRQoL for affected patients (67).

Moreover, the discrepancies observed between the Ethiopian and Zimbabwean value sets show the significance of using localized value sets for HRQoL assessments. Studies emphasized the importance of developing country-specific value sets that take into account local population characteristics, cultural factors, and healthcare systems (68). The fact that the Zimbabwean value set produces different utility scores for the same health states, when compared to the Ethiopian value set, shows the contextual differences in how health is perceived and valued. Similar discrepancies have been reported between value sets from different countries (12), further showing the necessity of utilizing context-specific tools in assessing the actual burden of diseases on populations (69).

In conclusion, this systematic review supports the growing body of evidence indicating that chronic diseases significantly lower HRQoL, and it emphasizes the importance of using country-specific value sets, like the Ethiopian EQ-5D-5l, to better capture the health burden within local contexts. This enhances the global understanding of the significant impact of chronic diseases and emphasizes the value of accurate, localized measurements of HRQoL to inform healthcare decision-making and policy interventions designed to specific regions and populations.

### Limitations and future directions

Several limitations should be considered when interpreting the results of this meta-analysis. First, the inclusion of studies using both EQ-5D-5l and EQ-5D-3l versions without applying conversion formulas may have introduced heterogeneity in utility values. Although conversion formulas are available, their accuracy across different populations may be limited. Second, the predominance of moderate-quality studies limits the robustness of some findings. Third, the geographical concentration of studies in urban settings, especially in Addis Ababa, may not fully represent health outcomes in rural areas, which may exhibit significant disparities. Additionally, the limited number of studies prevented subgroup or sensitivity analyses, particularly for specific conditions such as diabetes mellitus and HIV/AIDS. Finally, the assessment of quality of life in the context of diseases like HIV, which can vary significantly due to complications, presents additional challenges.

The restriction to certain databases may have introduced selection bias, potentially missing out on relevant studies published in regional journals. Additionally, while a thorough search strategy was implemented, hand-searching may have missed some relevant gray literature. There is also the potential for publication bias, as studies with significant findings are more likely to be published, which could bias the results. Finally, the risk of bias across the included studies, with one study rated as low quality, might have affected the overall findings and their interpretation.

The results of this review suggest critical implications for clinical practice and healthcare policy in Ethiopia. Understanding the disease-specific burden on HRQoL can help healthcare providers in prioritizing interventions, particularly for chronic conditions like diabetes, HIV/AIDS, and cancer. The frequent reporting of pain/discomfort and anxiety/depression as major dimensions affecting quality of life across diseases indicates a need for integrated mental health and pain management services in the healthcare system. The review suggests the need for further investment in value set development to represent the detailed health states of Ethiopian populations. Additionally, future research should focus on longitudinal studies to track changes in HRQoL over time and across different demographic groups. There is also a need for studies that include rural populations to ensure that healthcare interventions are equitable and reach those most in need. Future research could also explore the adaptation and validation of the EQ-5D tool for additional regional contexts in Ethiopia to enhance its utility in HRQoL assessments.

## Data Availability

The original contributions presented in the study are included in the article/Supplementary Material, further inquiries can be directed to the corresponding author.
