# Supplementary material for: Evaluating health-related quality of life in Ethiopia: systematic review and meta-analysis of EQ-5D-based studies
Source: Front Epidemiol. 2024 Nov 1;4:1455822. doi: 10.3389/fepid.2024.1455822 (PMC11563791; doi:10.3389/fepid.2024.1455822)
Supplement: Supplementary file 1 [file Datasheet1.pdf]

S2 Table: Search strategies and results for all databases		
**PubMed**		**Results**
**Search terms**	"Quality of life"[Text Word] OR "QoL"[Text Word] OR (Quality of life[MeSH Terms]) OR (QoL[MeSH Terms]) OR (HRQoL[Text Word]) OR (HRQoL[MeSH Terms]) AND (EQ-5D[Text Word]) OR (EQ-5D-5L[Text Word]) OR (EQ-5D-3L[Text Word]) OR (EuroQoL[Text Word]) OR ((EQ-5D[MeSH Terms]) OR (EQ-5D-5L[MeSH Terms]) OR (EQ-5D-3L[MeSH Terms]) AND (EuroQoL[MeSH Terms]) OR (five dimensions[MeSH Terms])) AND (((("Ethiopia"[MeSH] OR Ethiopia*[tw]	225
**Embase**		**Results**
**Search terms**	(Quality of Life/exp OR QoL/exp OR HRQoL/exp OR EQ-5D/exp OR EQ-5D-3L/exp OR EQ-5D-5L/exp OR EuroQol/exp OR "five dimensions"/exp OR Ethiopia/exp) OR ("quality of life" OR QoL OR "health-related quality of life" OR HRQoL OR EQ-5D OR EQ-5D-3L OR EQ-5D-5L OR EuroQol OR "five dimensions" OR Ethiopia) [tiab]	313
**Scopus**		**Results**
**Search terms**	(TITLE-ABS-KEY("Quality of life ") OR TITLE-ABS-KEY(“QoL”) OR (TITLE-ABS-KEY("HRQoL")) AND TITLE-ABS-KEY("EQ-5D") OR TITLE-ABS-KEY("EQ-5D-5L") OR TITLE-ABS-KEY("EQ-5D-3L") OR (TITLE-ABS-KEY("EuroQoL") OR TITLE-ABS-KEY“five dimensions")) AND TITLE-ABS-KEY("Ethiopia")	164
**Additional records identified through hand searching and other sources**		**Results**
**Search**	“Quality of life” OR “QoL” OR “HRQoL” AND "EQ-5D” OR “EQ-5D-5L”	123

**terms**	OR “EQ-5D-3L” OR “EuroQoL” OR “five dimensions" AND "Ethiopia" site:gov OR site:org OR site:ac	
**Total**		**825**
