# Supplementary material for: Evaluating health-related quality of life in Ethiopia: systematic review and meta-analysis of EQ-5D-based studies
Source: Front Epidemiol. 2024 Nov 1;4:1455822. doi: 10.3389/fepid.2024.1455822 (PMC11563791; doi:10.3389/fepid.2024.1455822)
Supplement: Supplementary file 2 [file Datasheet2.pdf]

S3 Table: Agency for Healthcare Research and Quality (AHRQ) checklist to assess quality of the included studies

ARHQ Methodology Checklist for Cross-sectional study	Sibhat et al., 2019	Araya et al., 2020	Kaso et al. 2022	Tito et al., 2022	Belachew and Sendekie., 2023	Sendekie AK, et al., 2023	Negash et al., 2023	Gebremariam GT et al., 2022	Iyar et al., 2024	Belay et al., 2021	Kaso et al. 2021	Kalayou Haftu et al., 2022	Tegegne., 2023	Shimeles et al., 2022
1. Define source of information (survey, record review)	1	1	1	1	1	1	1	1	1	1	1	0	1	0
2. List inclusion and exclusion criteria for exposed and unexposed subjects (cases and controls) or refer to previous publications	1	1	1	1	1	1	1	1	1	1	1	1	0	1
3. Indicate time period used for identifying patients	1	1	1	1	1	1	1	1	1	1	1	1	1	1
4. Indicate whether or not subjects were consecutive if not population-based	1	1	0	1	1	1	1	1	1	0	0	0	1	0
5. Indicate if evaluators of subjective components of study were masked to other aspects of	1	0	1	1	0	0	0	1	1	0	0	0	0	1

the status of the participants														
6. Describe any assessments undertaken for quality assurance purposes (e.g., test/retest of primary outcome measurements)	1	1	1	1	1	1	1	1	1	1	0	1	0	1
7. Explain any patient exclusions from analysis	0	0	0	0	0	0	0	1	0	0	0	0	0	0
8. Describe how confounding was assessed and/or controlled	1	1	0	1	1	1	1	1	1	0	1	1	0	0
9. If applicable, explain how missing data were handled in the analysis	0	0	0	0	0	0	0	0	0	0	0	0	0	0
10. Summarize patient response rates and completeness of data collection	1	0	1	1	1	1	1	1	0	0	0	1	0	0
11. Clarify what follow-up, if any, was expected and the percentage of patients for which incomplete data or follow-up was obtained	0	0	0	0	0	0	0	0	0	0	0	0	0	0
**Total score**	**8**	**6**	**6**	**8**	**7**	**7**	**7**	**9**	**7**	**4**	**4**	**5**	**3**	**4**

Quality Rating (High, Moderate, or High), 1 = yes, 0 = others (No, NA = not applicable, U = unclear)
